# Prognostic significance of combined radiologic imaging modalities for prognosis of clinical IA adenocarcinomas

**DOI:** 10.18632/oncotarget.23395

**Published:** 2017-12-18

**Authors:** Hiroaki Kuroda, Shnsuke Mori, Hirotaka Tanaka, Tatsuya Yoshida, Tetsuya Mizuno, Noriaki Sakakura, Yasushi Yatabe, Hiroshi Iwata, Yukinori Sakao

**Affiliations:** ^1^ Department of Thoracic Surgery, Aichi Cancer Center Hospital, Aichi, Japan; ^2^ Department of Pathology and Molecular Diagnostics, Aichi Cancer Center Hospital, Aichi, Japan; ^3^ East Nagoya Radiological Diagnosis Foundation, Aichi, Japan; ^4^ Department of Thoracic Oncology, Aichi Cancer Center Hospital, Aichi, Japan

**Keywords:** three-dimensional, mediastinal size, invasive size, positron emission tomography, adenocarcinoma

## Abstract

**Background:**

We previously proposed measuring tumor size using mediastinal window setting on high-resolution computed tomography (CT) as a simple and useful modality for preoperative prognostication of small adenocarcinoma. Hence, the importance of tumor volume and positron emission tomography (PET) for preoperative prognostication of clinical stage IA (cIA) adenocarcinoma was studied.

**Materials and Methods:**

We retrospectively evaluated total 324 patients who underwent pulmonary resection of cIA adenocarcinoma between July 2008 and August 2015. Reconstructed three-dimensional (3D) images from 1–1.5 mm-sliced CT were evaluated for whole tumor volume including ground grass opacity, consolidation volume on lung window setting, and mediastinal window volume (MWV). The values examined by PET were total lesion glycolysis (TLG), and maximum standardized uptake (SUV max) and mean. Pathologic status was evaluated according to tumor maximum size, invasive size (IS), lymphatic and vascular vessels, pleural invasion (ly/v/pl), and TNM staging.

**Results:**

According to ly/v/pl invasion and lymph node positivity, no variables were superior to IS. We used Mean/MWV (SUV mean x MWV) to evaluate tumor quality and quantity in the role of surrogate TLG. Mean/MWV were superior to IS. Additionally, Mean/MV was associated with lymph node metastases. Among the various histologic subtypes, solid-predominant had the highest expression of Mean/MV. The higher Mean/MV significantly contributed to unfavorable disease-free survival in cIA adenocarcinomas, but not to overall survival.

**Conclusions:**

The mean/MWV value determination on 3D-reconstructed CT images was a simple and useful preoperative modality for predicting invasive facet in cIA adenocarcinoma. However, higher values didn't significantly affect overall survival.

## INTRODUCTION

The correlation between tumor proliferation and standardized uptake value (SUV) on positron emission tomography (PET) was observed in non-small cell lung cancer (NSCLC) [1, 2]. However, the degree of ^18^F-fluorodeoxyglucose (FDG) uptake does not always predict prognosis [2, 3]. By combining with lung tumor size of > 3 cm, SUV can help identify NSCLC patients with poor prognosis [4]. Total lesion glycolysis (TLG) is the multiplication of mean SUV and metabolic tumor volume (MTV). Chen et al. commented that the summation of whole-body tumor glycolysis, represented by the whole-body TLG, may indicate tumor burden and be a good indicator of prognosis [5]. Other studies suggested that MTV and TLG are better prognostic measures than maximum SUV (SUV max) and mean SUV (SUV mean) for early- and advanced-stage NSCLCs, respectively [3, 6, 7].

The invasive component of a lesion has been known to histopathologically correspond to tumor aggressiveness [8]. Specifically, the solid component of a lesion is recognized as the invasive area or scar, which excludes the lepidic component in lepidic predominant adenocarcinoma; these findings can be easily defined using lung window or mediastinal window settings on computed tomography (CT) [9, 10]. Moreover, preoperative evaluation of tumor size by measurement of mediastinal diameter (MD) on CT may predict prognosis, lymph node metastasis, and tumor invasiveness of small adenocarcinomas (1–3 cm) or clinically early-stage tumors [10]. Various studies have documented the correlation between CT findings and the pathological features of lung adenocarcinoma [9, 10]. However, several authors reported that the tumor volume was a prognostic indicator for NSCLC [11, 12]. In fact, because lung cancers having a longer cephalocaudal diameter than axial one sometimes exists on CT sagittal view, it is hard to confirm that it is evaluated precisely. Hence, we intended to use the index of Mean/MWV (SUV mean × MWV) to substitute for TLG in evaluating quality and quantity simultaneously, which was compatible with precise total lesion glycolysis. This is because thin slice CT can measure exact volume than the rough image of CT in PET.

**Figure 1:**
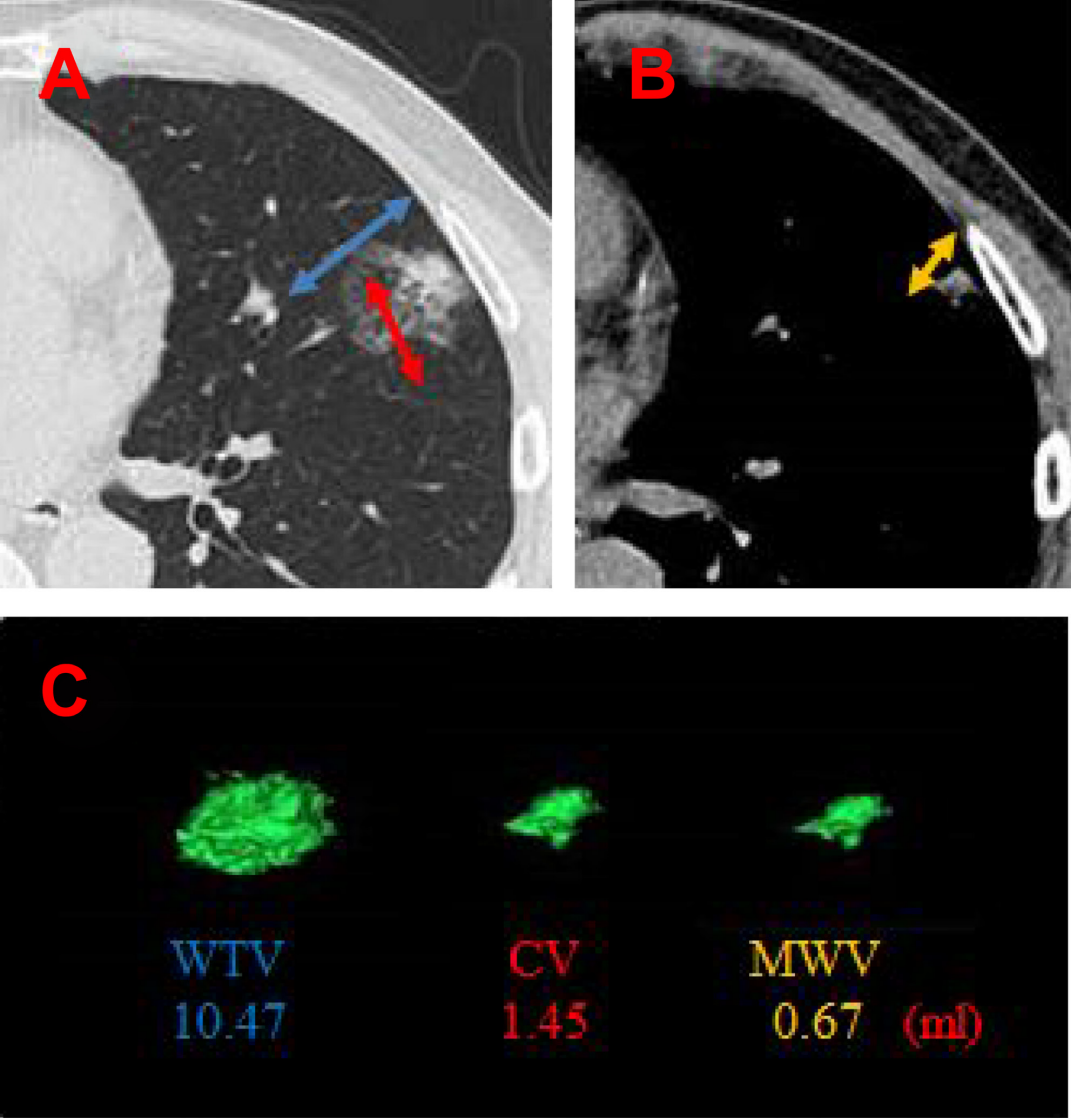
Three-dimensional reconstructed images of a representative pulmonary adenocarcinoma (**A**) Ground grass opacity lesion (Blue) and consolidation (Red) on lung window setting. (**B**) Mediastinal size on mediastinal window setteing (Yellow). (**C**) Each reconstructed schema and value (ml).(CV: Consolidation volume; MWV: Mediastinal window volume).

In this study, we evaluated total 324 lung adenocarcinomas from single institution between July 2008 and August 2015, and divided into two groups. During this time, the concept of invasive size is announced, and this is because the pathological evaluation method changed. The majority of this study aimed to clarify the most efficacy of the tumor volume evaluated by combination of three-dimensional (3D) reconstructed CT image modalities [Whole Tumor Volume, Consolidation Volume, and Mediastinal Window Volume (MWV)] (Figure 1), 2D conventional axial view modalities [Maximum tumor diameter, Consolidation diameter, and MD], PET as a predictive factor of prognosis (SUV max, mean and TLG), and tumor marker (CEA) against biologic aggressiveness such as lymphatic vessel, vascular vessel, or pleural invasion and pathological lymph node positive compared with pathological invasive size prior to surgical resection of clinical stage IA (cIA) adenocarcinomas. Finally, we evaluated the prognosis of 106 cIA adenocarcinomas before the establishment of the concept of pathological invasive size to dissolve one question why association with biologic aggressiveness is important above and beyond disease free survival (DFS) and overall survival (OS)?

## RESULTS

The baseline characteristics of the study population (*n* = 324) are summarized in Table 1. The first subgroup of cIA adenocarcinomas comprised 180 women and 144 men, with a median age of 66 years (range 32–86 years) who underwent pulmonary resection after the establishment of the concept of pathological invasive size and predominant histological subtypes. Representative CT images, and the reconstructed volumes and values were shown in Figure 1.

**Table 1:** Characteristics of patients with clinical stage IA adenocarcinomas

	cIA patients (*n* = 324)
Age (years)	
Median (range)	66 (32–86)
Sex	
Male/Female	144/180
cT status	
cT1a	96
cT1b	116
cT1c	112
Histology	
Minimally invasive carcinoma	42
Invasive adenocarcinoma	282
pT status	
pT1a(mi)/T1a	42/57
pT1b	123
pT1c	32
pT2a	66
pT3	4
pN status	
pN0	284
pN1	19
pN2	21
Pathological stage	
pIA1/pIA2/pIA3	95/115/29
pIB	43
pIIB	20
pIIIA	22
Genomic mutations	
EGFR / non-EGFR	180 / 144
KRAS / non-KRAS	31 / 293
ALK / non- ALK / unknown	10 /280 / 34
Triple negative / unknown	71 / 34

### Preoperative, postoperative and the index of Mean/MWV factors associated with lymphatic vessel, vascular vessel, or pleural invasion and pathological lymph node postive in cIA adenocarcinomas

As shown in Table 2, among the variables such as 2D images (Maximum tumor diameter, Consolidation diameter, and MD), 3D images (Whole tumor volume, Consolidation volume, and MWV), preoperative PET (SUV max and TLG), and tumor marker (CEA), there were no factors superior to pathological invasive size (IS) (0.81 and 0.78) in the area under the curve (AUC) for both lymphatic/vascular/pleural (ly/v/pl) invasion and lymph node positivity on receiver operating characteristic (ROC). However, the index of Mean/MWV (0.82 and 0.84) was superior to IS for the assessment of cIA adenocarcinomas.

**Table 2:** The AUC values for invasive characteristics in clinical stage IA adenocarcinomas

Variables	Ly/v/pl invasion AUC (95% CI)	LN positive AUC (95% CI)
2D imaging		
Tumor maximum diameter	0.59 (0.52–0.67)	0.65 (0.53–0.78)
Consolidation diameter	0.76 (0.69–0.82)	**0.80 (0.72–0.89**)
MD	0.79 (0.73–0.85)	**0.83 (0.76–0.91)**
3D imaging		
Whole tumor volume	0.62 (0.54–0.69)	0.66 (0.54–0.79)
Consolidation volume	0.78 (0.72–0.85)	**0.81 (0.73–0.89)**
MWV	0.80 (0.73–0.86)	**0.82 (0.74–0.90)**
Preoperative PET		
SUV max	0.78 (0.72–0.84)	**0.80 (0.70–0.91)**
TLG	0.71 (0.64–0.77)	0.72 (0.61–0.84)
Tumor marker		
CEA	0.57 (0.49–0.64)	0.52 (0.38–0.66)
Pathological diameter		
Tumor maximum size	0.62 (0.55–0.70)	0.64 (0.51–0.77)
**IS**	**0.81 (0.75–0.87)**	**0.78 (0.69–0.88)**
Volume and PET		
Mean/MWV	**0.82 (0.76–0.87)**	**0.84 (0.76–0.92)**

characteristics in clinical stage IA adenocarcinomas AUC, area under the curve; LN, CEA, carcinoembryonic antigen; MD, mediastinal diameter, MWV, mediastinal window volume; PET, positron emission tomography; SUV, standardized uptake value; TLG, total lesion glycolysis; 2 or 3D, two- or three-dimensional.

### The index of mean/MV associated with lymph node metastasis

**Figure 2:**
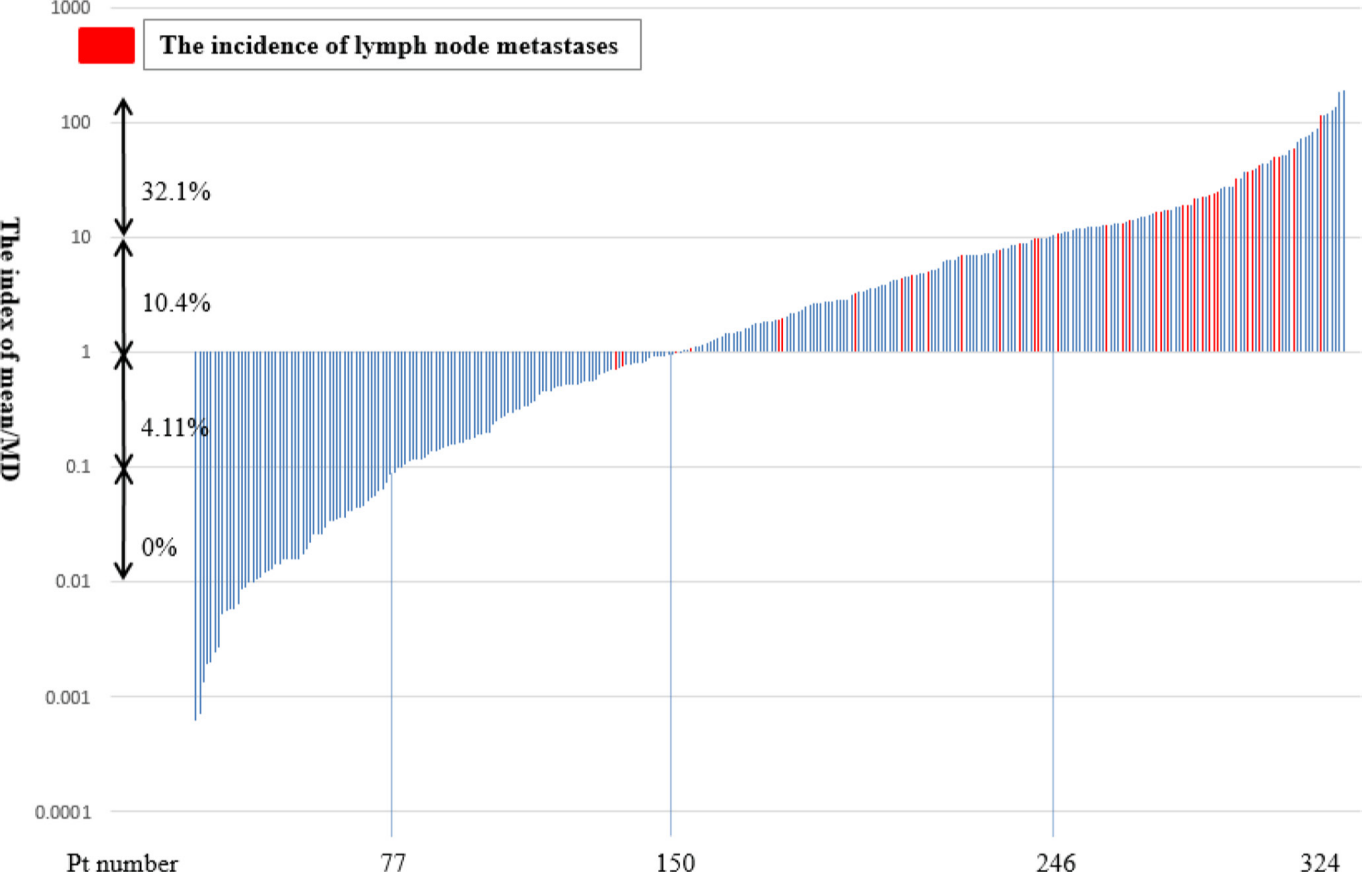
Incidence of lymph node metastasis according to the index of mean/mediastinal window volume (MWV) A red bar showed a patient with hilar and/or mediastinal lymph node metastasis and a blue bar showed a patient without lymph node metastasis.

The incidence of lymph node metastases in cIA NSCLC was 12.3% (40/324) (Table 1). According to the index of mean/MWV, the incidence was 0% for ≤ 0.10 (*n* = 77), 4.11% for 0.11–1.00 (*n* = 73), 10.4% for 1.01–10.00 (*n* = 96), and 32.1% for > 10.00 (*n* = 78) (Figure 2).

### The association of the index of Mean/MWV with the predominant histologic subtype and genomic mutation in cIA adenocarcinoma

**Figure 3:**
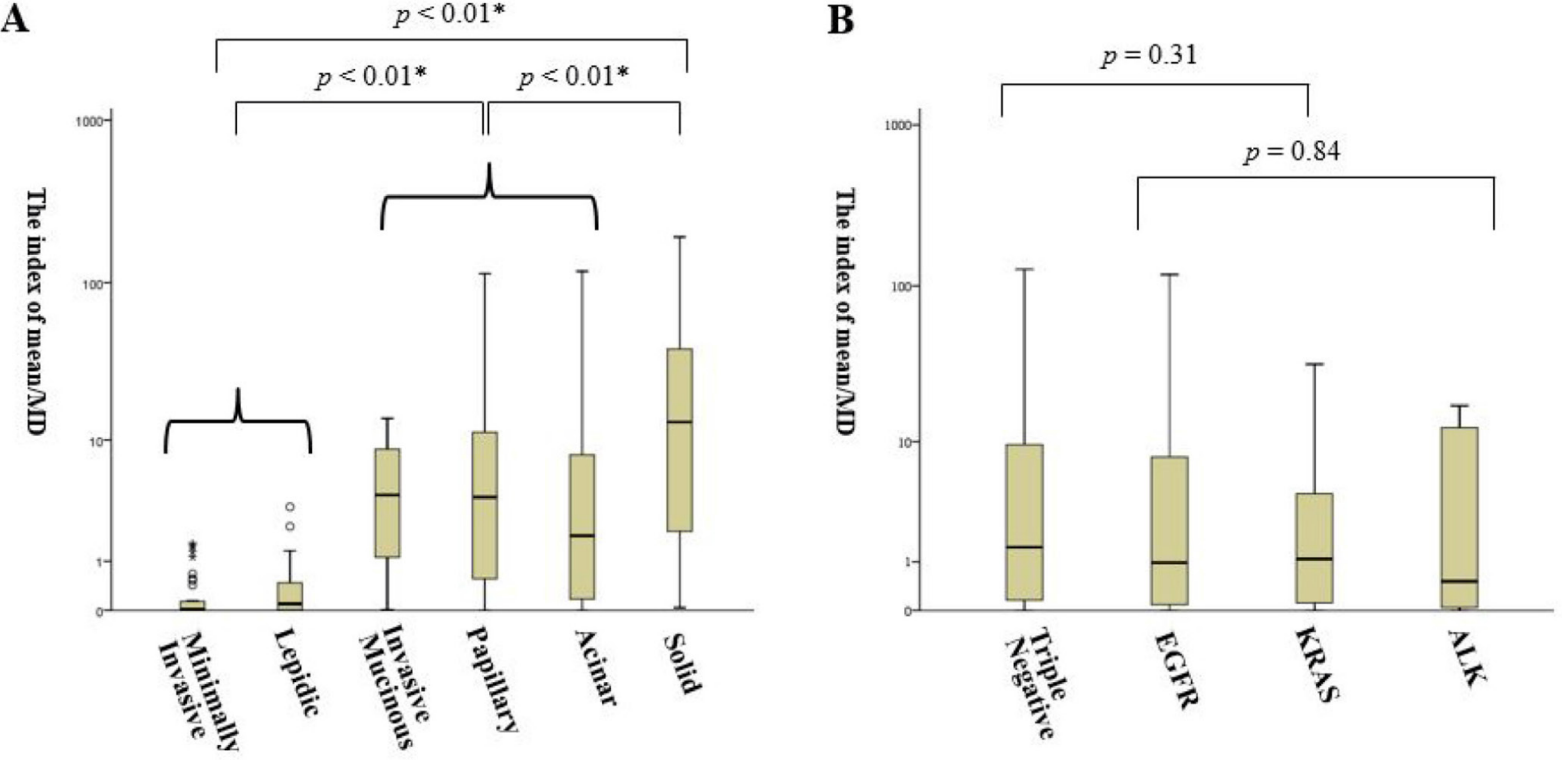
The index of mean/mediastinal volume in clinical stage IA adenocarcinoma (**A**) Analysis by the predominant histologic subtypes. Comparison among minimally-invasive and lepidic (left); invasive mucinous, papillary, and acinar (middle); and solid (right) types is shown. (**B**) Analysis by the classification of genetic mutations. Comparison among triple negative and mutation groups (EGFR, KRAS, or ALK). ^*^p < 0.05.

We examined whether the index of Mean/MWV was associated with histologic subtype and genomic mutations in small adenocarcinoma. The highest value for the index of Mean/MWV in the predominant histologic subtypes were identified in solid (36.4 ± 50.8, *n* = 36), followed by papillary (11.6 ± 20.6, *n* = 113), papillary (7.2 ± 15.5, *n* = 85), invasive mucinous (9.9 ± 18.1, *n* = 16), lepidic (0.42 ± 0.72, *n* = 32), and minimally-invasive adenocarcinoma (MIA) (0.39 ± 1.05, *n* = 42) (Figure 3A). The value was significantly higher in solid than in acinar/papillary/invasive mucinous, lepidic types, and minimally invasive adenocarcinoma (*p* < 0.01) (Figure 3A). The value in triple negative mutation adenocarcinoma (10.5 ± 22.6, *n* = 71) was higher by 1.3-fold than that in EGFR-, KRAS-, or ALK-positive adenocarcinoma (8.0 ± 17.9, *n* = 219), but no significant difference was found (*p* = 0.31) (Figure 3B). Additionally, there was no significant difference between the mutation-positive types [EGFR (8.9 ± 19.5, *n* = 177) vs. KRAS (4.0 ± 7.0, *n* = 32) vs. ALK (5.0 ± 7.4, *n* = 10); *p* = 0.84] (Figure 3B).

### The association of the index of Mean/MWV with patient outcomes

**Figure 4:**
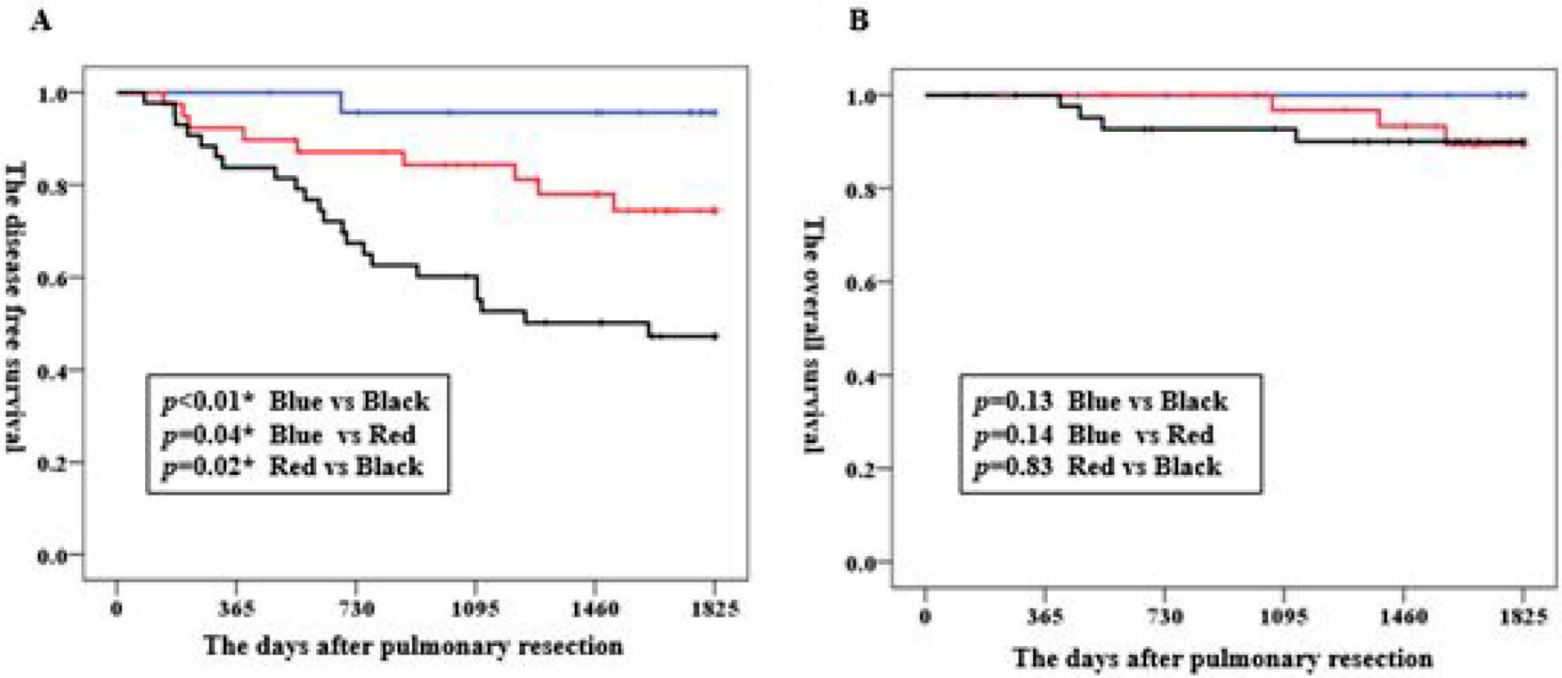
Kaplan–Meier graphs of the disease-free survival (A) and overall survival (B) in patients with clinical stage IA adenocarcinoma Blue line, low-expressing group (≤ 1; *n* = 24); Red, medium-expressing group (1.1 to 10; *n* = 39); Black line, high-expressing group (> 10; *n* = 43). ^*^p < 0.05.

The second group consists of 106 consecutive surgically resected adenocarcinomas that were subjected to evaluation of the relationship between the index of Mean/MWV and disease-free survival (DFS) after operation and overall survival (OS), patients with 1779 days in median following course who underwent pulmonary resection before the establishment of the concept of pathological invasive size and predominant histological subtypes were analyzed by classifying them to the low-expressing group (≤ 1; *n* = 24), medium-expressing group (1.1 to 10; *n* = 39) and high-expressing group (> 10; *n* = 43). As shown in Figure 4A, DFS was significantly lower in the high-expressing group than in the low-expressing group (*p* < 0.01) and the medium-expressing group (*p* = 0.02). OS in the high-expressing group showed a tendency for a poorer prognosis, compared with the low-expressing (*p* = 0.13) and medium-expressing (*p* = 0.83) groups (Figure 4B).

## DISCUSSION

In this study, the efficiency of all 3D measurements, to detect the invasiveness of cIA adenocarcinomas was inferior to pathological invasive size; quantitative variables such as SUV max, TLG, and CEA were also inferior or equivalent to the pathological assessment of invasiveness. Travis reported that in early-stage tumors, the measurement of tumor size for staging may need to take into account only the size of the invasive component, instead of the tumor as a whole [2, 3, 10]. Invasive tumor size is an independent prognostic factor that may be better than overall tumor size in predicting the outcomes of lepidic predominant tumors [8, 13]. To overcome these disadvantages of TLG and MD in precisely calculating the true invasive component of a tumor, we used MWV as a substitute for MTV. However, the value of these indices of volume-dependent parameters and qualitative factors for both clinically early and advanced NSCLC has not been published. Our measurement of the index of Mean/MWV seemed to correspond to tumor malignant behavior and prognosis.

One main reason exists why we used MWV as a substitute for MTV. Our previously reports suggested that tumor dimension determined by mediastinal window settings provided additional useful prognostic data that could not be evaluated by lung window settings in previous studies [9, 10, 14]. Furthermore, tumor size was a significantly better prognostic factor when evaluated by MD, instead of consolidation maximum diameter on lung window setteing. MD was an important predictive factor for prognosis as well as for lymph node involvement and tumor invasiveness in small (1–3 cm) lung adenocarcinoma [10]. Additionally, the high correlation between IS and MD findings were previously postulated [14]. Furthermore, for the evaluation of the tumor malignant behavior, the mediastinal window setting reflected it among various imaging settings, and we considered that even volume (3D) was similar to the area (2D).

Several authors have reported that SUV max was a significant prognostic factor in NSCLC [15, 16]. Interpreting SUV max has been and will probably continue to be challenging and controversial because the optimal FDG has not been defined. FDG uptake in tumors is proportional to the metabolic activity of viable cells, and therefore, may help predict biologic aggressiveness [1–3]. However, several authors have reported that TLG and MTV were better than SUV max in the prognosis of NSCLC [3, 15, 16]. TLG is an established and important prognostic factor. In addition, Park et al. recently reported that TLG was a significant prognostic factor for OS in patients with stage IA NSCLC [17]. Furthermore, a combination of MTV and TLG, according to TNM stage, was reported to be predictive of patient survival independent of clinical stage [2, 16]. Park et al. indicated three well-known limitations of SUV max: 1) it largely depends on many biologic factors (body weight, serum glucose level, etc.) and technical factors (interscanner variability, image reconstruction method, etc.); 2) it does not take into account tumor volume, which is a well-known prognostic determinant; and 3) it measures a single highly-metabolic focus that may not accurately reflect the metabolic activity of the whole tumor [17]. In this study, SUV max was superior to TLG in the assessment of biologic aggressiveness, such as lymph node metastases and ly/v/pl invasion. These findings may be because the patient population was limited to those with cIA and the low incidence (13.0%) of patients with non-invasive cancer. Metabolic volume measurement by PET CT seemed to be relatively difficult. Therefore, evaluation using CT may calculate a more accurate value, particularly in cIA NSCLC.

Several authors have reported that larger or more desmoplastic fibrous scars were associated with more aggressive tumor invasion and poorer prognoses [13, 18, 19]. In other words, measuring the size of the solid component, defined as the lesion without GGO on lung window setting, is a promising method of determining the specific invasive site (scar) of the adenocarcinoma. Our study suggested that solid-predominant tumors had the highest expression of Mean/MWV among the various histologic subtypes, but the number of genomic triple negative adenocarcinoma was not significantly higher than that of adenocarcinoma harboring exclusive gene of either EGFR, KRAS, or ALK. In addition, the index of Mean/MWV was a strong predictor of DFS, even at cIA stage. Yanagawa et al. reported that solid predominant and invasive tumor size were independent predictors of increased risk of recurrence in 191 stage I adenocarcinomas [20], which is compatible with our new strategy ‘Mean/MWV’.

This study has several limitations. First, we used two different cohorts in this study. We used the first cohort after a concept of invasive size announced for evaluation of invasiveness and malignant behavior which were defined by pathological features, not prognosis. Assessment of the robustness of these characteristics of tumor aggressiveness requires long-term follow-up, particularly in cIA NSCLC. In this study, we evaluated and discussed prognosis by using another cohort before the announcement of a concept of invasive size. This provide an image comparator by which evaluation value and an evaluated reproduced image can be viewed without any reevaluating pathological subtypes and the relationship between the evaluation value and prognosis could purely be recognized; however, the divided two cohorts made huge bias. Second, although the number of the study population was large, the retrospective and single-center design may have added bias to the measurement of invasive size by the same strategy for diagnosis. Third, the measurement error should be considered in PET evaluation. Biehl et al reported that the choice of threshold will influence the measurement of tumor volume, SUV mean, and whole-body TLG and that no single optimal threshold can provide accurate tumor delineation [21]. It is important to note that SUV threshold may differ among facilities, but the strong point of this cohort was the large sample size to minimize the errors that may occur by single-institutional PET evaluation. However, future prospective studies, particularly for the planning phase, are still needed to validate our results. Fourth, MD may have been overestimated in this study; nevertheless, the index of Mean/MV proved to be a good indicator of biological behavior in this study.

In conclusion, the index of Mean/MWV on 3D reconstruction of high-resolution CT images was simple and may be a useful preoperative modality for dividing two pathological groups between minimally invasive adenocarcinoma and invasive adenocarcinoma. Higher values was associated with the incidence of lymph node metastasis, and significantly contributed to unfavorable disease-free survival in cIA adenocarcinomas, but not to overall survival in cIA adenocarcinomas.

## MATERIALS AND METHODS

This was a retrospective study on 324 patients who underwent surgical resection for preoperative cIA NSCLC at the Aichi Cancer Center between July 2008 and August 2015. Tumor dimension was evaluated under two different CT imaging conditions: 1) lung setting [level = 2500 Hounsfield unit (HU), width = 1500 HU] and 2) mediastinal setting (level = 60 HU, width = 350 HU). The 3D reconstructed 1–1.5 mm-slice images of the whole tumor based on thin-sliced CT (AZE Virtual Place; AZE, Tokyo, Japan) were evaluated and calculated factors such as whole tumor volume, consolidation volume, and MWV (Figure 1).

For all patients, preoperative evaluation for distant metastasis was assessed using chest CT, abdominal CT or ultrasonography, brain CT or magnetic resonance imaging, and PET. Variables such as TLG, SUV max, and SUV mean were obtained by PET; TLG was calculated as the product of SUV mean and MTV. We intended to use only the PET images photographed at East Nagoya Radiological Diagnosis Foundation to fix the dispersion of the value between facilities. Clinically, mediastinal and hilar lymph node status was deemed positive if the chest CT findings revealed a lymph node short axis size of at least 1.0 cm; nodal stage was assigned according to the 8th Edition of the TNM Classification of Malignant Tumors [22]. Histopathological diagnosis was based on the 2004 World Health Organization classification [23]. The 3D volume was calculated by one independent radiological technologist who was blinded to the clinical information of the patients; this calculation was reviewed by three thoracic specialists, including one thoracic oncologist and two thoracic surgeons.

Patient records were examined for age, gender, preoperative serum CEA levels, and clinical TNM staging. Pathological stage was evaluated by pathological tumor maximum size; IS; ly/v/pl invasion; and pathological TNM staging. Because individual patients were not identified, our institutional review board approved this study without the requirement to obtain written informed consent.

We additionally evaluated 106 (32.5%) in the analysis of prognosis between July 2008 and December 2011based on before the establishment of the concept of pathological invasive size and predominant histological subtypes in adenocarcinomas [8, 13]. The patient records and information were made anonymous prior to analysis. Finally, patients who were diagnosed as having adenocarcinoma and whose median clinical course was monitored for 1779 days were enrolled to evaluate whether the modalities could reflect postoperative prognosis.

### Statistical analyses

All data were analyzed using the Statistical Package for the Social Sciences (SPSS) version 17.0 (SPSS Institute Incorporated; Chicago, Illinois, USA). ROC analyses were performed for several variables, and AUC was calculated. Differences between two groups were calculated using the Mann–Whitney *U* test; comparisons among groups of more than three were calculated using the Kruskal–Wallis test. A *p* value of < 0.05 was considered to indicate statistical significance.
